# Obstructive shock due to tracheal perforation following long-term placement of a tracheostomy tube in a pediatric patient: a case report

**DOI:** 10.1186/s40981-022-00522-7

**Published:** 2022-04-21

**Authors:** Wataru Sakai, Yuko Nawa, Oba Junichi

**Affiliations:** 1 Pediatric Intensive Care Unit, Hokkaido Medical Center for Child Health and Rehabilitation, Sapporo, Hokkaido 006-0041 Japan; 2 Department of Anesthesiology, Sapporo Medical University School of Medicine, Sapporo, Japan

**Keywords:** Tracheal perforation, Pneumomediastinum, Obstructive shock

## Abstract

**Background:**

Tracheal perforation, although rare, is a known late complication of tracheostomy tube placement.

**Case presentation:**

We present a 7-year-old boy with severe physical and mental disabilities under tracheostomy and long-term mechanical ventilation and steroid therapy who suddenly developed obstructive shock secondary to pneumomediastinum and pneumothorax. Prior bronchoscopy had shown the tip of the tracheostomy tube contacting the posterior tracheal wall, causing ulceration and subsequent tracheal perforation. The perforation was bridged using a cuffed tracheostomy tube, but the patient subsequently died of additional comorbidities.

**Conclusions:**

Our experience suggests that tracheal perforation should be considered when pediatric patients with tracheostomy tubes suddenly develop hypotension.

**Supplementary Information:**

The online version contains supplementary material available at 10.1186/s40981-022-00522-7.

## Background

Iatrogenic or traumatic tracheal perforation is a rare condition, and the causes include early or delayed tracheal injury secondary to endotracheal intubation, tracheostomy tube placement, tracheostomy, and thyroid surgery [1]. Tracheal perforation can cause pneumomediastinum and pneumothorax, which can lead to obstructive shock requiring emergency treatment.

A poorly positioned tracheostomy tube might cause tracheal ulceration and perforation. Pressure exerted by the distal end of the tracheostomy tube impinging on the posterior tracheal wall and overinflation of the cuff can cause erosion of the posterior trachea [1]. Undernutrition, airway infection, hypotension, hypoxemia, anemia, diabetes, and steroid therapy are all known risk factors for tracheal perforation [2]. Although iatrogenic tracheal perforation with an endotracheal tube has been previously reported in several studies, reports of perforation due to a tracheostomy tube in pediatric patients are rare [3].

We experienced a case of sudden hypotension because of pneumomediastinum and pneumothorax secondary to tracheal perforation in a pediatric patient.

This report was approved by the Ethics Committee of the Hokkaido Medical Center for Child Health and Rehabilitation. Written consent was obtained from the patient’s parents for publishing this case report. This manuscript adheres to the CARE guideline.

## Case presentation

The patient was a 7-year-old boy under mechanical ventilation due to severe physical and mental disabilities secondary to accidental hypoxic-ischemic encephalopathy. Tracheostomy with an uncuffed tracheal tube for mechanical ventilation had been performed at the age of 10 months. The patient had been diagnosed with cerebral palsy and panhypopituitarism, leading to diabetes insipidus, central hypothyroidism, and secondary adrenocortical insufficiency, which required permanent steroid replacement therapy. X-ray examination showed scoliosis associated with cerebral palsy.

**Fig. 1:**
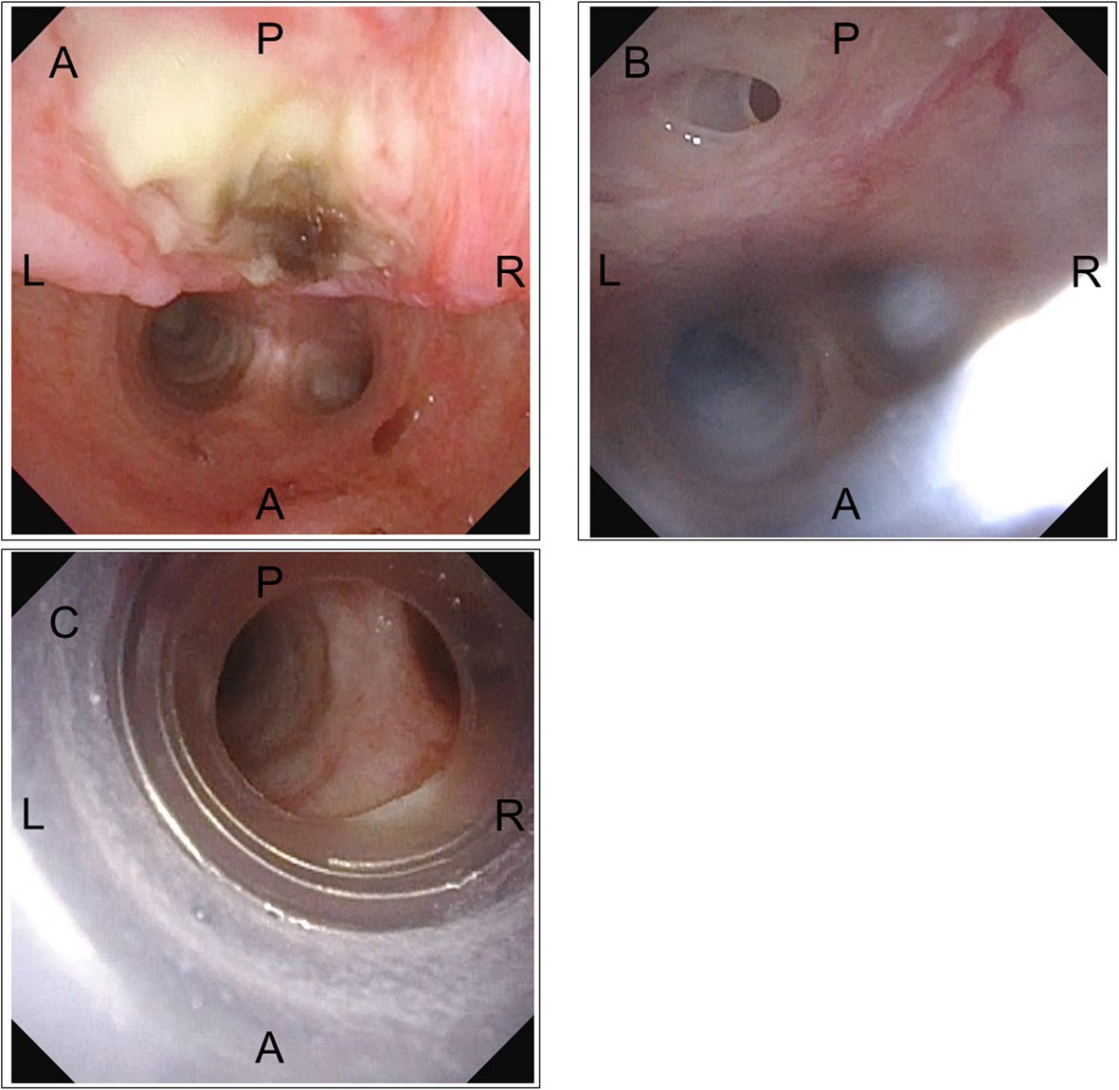
Bronchoscopy findings during the patient’s clinical course. **A** Ulceration of the posterior tracheal wall was observed on the day of admission. **B** Perforation of the posterior tracheal wall on day 3. **C** The tracheal perforation was bridged by a cuffed tracheostomy tube. A, anterior tracheal wall; P, posterior tracheal wall; L, left; R, right

Before his current presentation, massive pleural effusion due to sepsis had worsened his respiratory and physical status, requiring bilateral thoracic drainage tube insertion. Since the sepsis and massive pleural effusion could not be controlled at the previous hospital, he was transported to our hospital by a medical airplane and an ambulance, a journey which took 3 h. On his arrival at our hospital, otorhinolaryngologists performed bronchoscopy to verify the position of the uncuffed 6.5 mm silicon cannula PHL (KOKEN, Tokyo, Japan) because his tidal volume on mechanical ventilation was insufficient, which showed that the tip of the tracheostomy tube impinged on his posterior tracheal wall, as well as evidence of a tracheal ulcer (Fig. 1A and Video 1). The uncuffed tube was removed and replaced with a cuffed 6.0 mm GB Adjustfit® tube (Fuji Systems, Tokyo, Japan), using bronchoscopy to confirm that the tube did not contact the tracheal ulcer (Video 1), and the adequacy of tidal volume was confirmed. At this point, there was no tracheal perforation, as seen on bronchoscopy (Video 1).

**Fig. 2:**
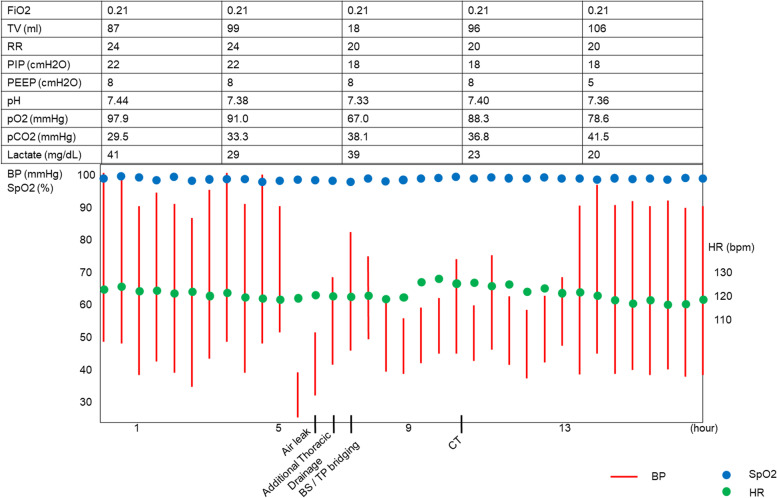
The patient’s clinical course on day 3. BP, blood pressure; HR, heart rate; SpO_2_, percutaneous oxygen saturation; BS, bronchoscopy; CT, computed tomography

**Fig. 3:**
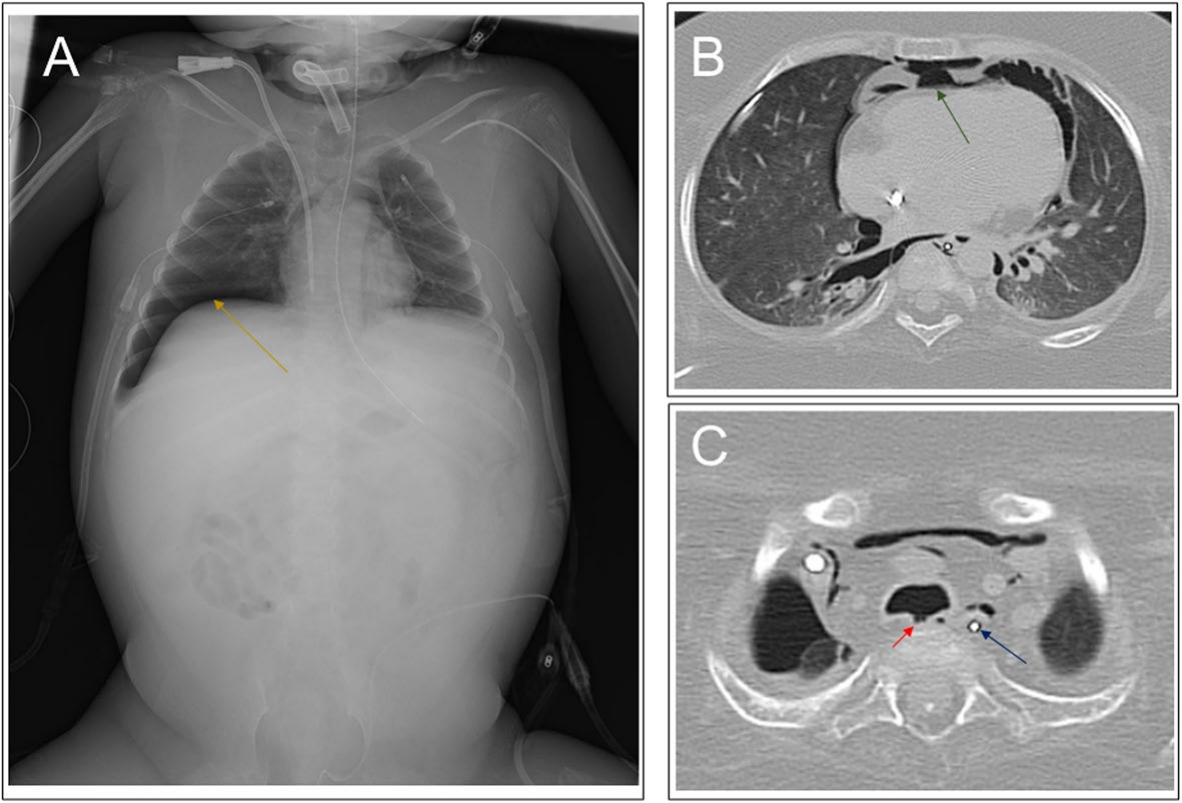
Chest X-ray and CT images showing pneumothorax, pneumomediastinum, and tracheal perforation. **A** Chest X-ray showed pneumothorax and pneumomediastinum before insertion of an additional thoracic drainage tube. Chest CT showed **B** pneumomediastinum and **C** tracheal perforation. The yellow, green, red, and blue arrows show pneumothorax, pneumomediastinum, the tracheal perforation, and esophagus, respectively

On day 3, the patient’s blood pressure suddenly became unstable, and air leakage from the right thoracic drainage tube was noticed, suggesting the presence of a pneumothorax (Fig. 2). Although auscultation showed diminished breath sounds in the right lung as compared to earlier examinations, tidal volume was maintained because of the presence of the previously placed right thoracic drainage tube, and the ventilator settings did not require changing (Fig. 2). However, his partial pressure of oxygen decreased from 91 to 67 mmHg (Fig. 2). Since his blood pressure dropped critically despite the administration of vasopressin and drainage of the pneumothorax via the chest drains (Fig. 3A), an 8-Fr Argyle™ trocar catheter (Covidien™, Tokyo, Japan) was inserted into his anterior chest. Subsequently, although the vasopressor dose could be decreased with additional thoracic drainage, his blood pressure did not recover completely. Since air leak from the newly inserted right thoracic drainage tube continued during both inspiration and expiration, tracheobronchial rupture rather than lung injury was suspected. Repeat bronchoscopy confirmed the presence of tracheal perforation at the site of the tracheal ulcer that had been observed on the day of admission (Fig. 1B and Video 1). Hence, we adjusted the cuff of the tracheostomy tube, positioning it distal to the tracheal perforation and, thus, bridging the perforation, ensuring that the tip of the tracheostomy tube no longer contacted his tracheal wall (Fig. 1C and Video 1). Chest computed tomography (CT) performed after the patient achieved hemodynamic stability revealed tracheal perforation, pneumomediastinum, and right pneumothorax (Fig. 3B). The tracheal perforation was at the level of the Th-3 vertebra, 20 mm cranial to the carina (Fig. 3C).

On day 8, the patient developed uncontrollable sepsis with worsening of laboratory data despite antibiotic therapy. He was given best supportive care due to his irreversible neurological prognosis and died on day 14.

## Discussion

Obstructive shock occurs due to great vessel or cardiac ventricular obstruction [4]. Several conditions can cause obstructive shock, such as tension pneumothorax, pneumomediastinum, cardiac tamponade, and pulmonary embolism [5]. The most common medical causes of pneumomediastinum are asthma exacerbation and infections [6]. Pneumomediastinum is treated with percutaneous mediastinal tube drainage or thoracotomy [7]. In our case, obstructive shock was caused by pneumothorax and pneumomediastinum secondary to tracheal perforation due to long-term tracheostomy tube placement. Although additional thoracic drainage restored hemodynamic stability, it took 8 h for the patient’s blood pressure to recover completely because the pneumomediastinum was treated conservatively by observation. However, since the pneumomediastinum did not worsen following bridging of the tracheal perforation by the cuffed tracheostomy tube, we considered it prudent to avoid aggressive intervention for the pneumomediastinum, due to the associated risks of bleeding, cardiac puncture, and infection.

Pediatric tracheal perforations can occur as both early and delayed complications of tracheostomy and endotracheal intubation [1, 2]. Injury to the posterior tracheal wall by an endotracheal tube is a known cause of tracheal perforation [8]. Long-term pressure on the posterior tracheal wall by the tip of the tracheostomy tube can lead to tracheo-esophageal fistula formation in children [1, 7]. Although rare, this complication is, at present, more common in immunocompromised children with poor healing who are tracheostomy dependent [1]. Additionally, children with tracheal anomalies and severe kyphoscoliosis have a greater risk of tracheoesophageal fistulas [1]. Our patient had severe scoliosis, and hence, his esophagus was significantly displaced to the left and was not directly behind the trachea (Fig. 3C). Since there was only soft tissue between the trachea and Th-3 vertebra, the shear stress on the soft tissue between the poorly positioned tip of the tracheostomy tube and Th-3 vertebra probably caused the perforation and subsequent pneumomediastinum and pneumothorax.

There were four possible reasons for the tracheal fragility in our case. First, our patient had cerebral palsy with undernutrition, long-term steroid replacement therapy, and severe sepsis because of an intractable cerebral abscess, making him a compromised host. Second, higher positive pressure ventilation was required during the course of treatment, which could have caused pressure injury. Use of an uncuffed tracheostomy tube during positive pressure ventilation is usually associated with some amount of air leak from the trachea. Changing to a cuffed tracheostomy tube without an air leak would result in higher pressure ventilation compared with an uncuffed tracheostomy tube with air leak despite unchanged positive pressure settings. Third, the unstable tip of the tracheostomy tube might have impinged on the posterior tracheal wall more strongly during the patient’s transfer to our hospital, which could have caused the wound that ultimately led to tracheal perforation. Fourth, it was possible that the cuff of the tracheostomy tube contacted the tracheal ulcer, causing the perforation, although we repeatedly confirmed that the cuff of the tracheostomy tube did not contact the tracheal ulcer during the course of treatment. Patients with a greater risk of tracheal perforation, such as our case, should undergo repeated assessment of the position of the tracheostomy tube not only during their hospitalization but also while being transferred, to prevent tracheal perforation.

The goal of treatment of tracheal perforation is to minimize the risk of mediastinitis and provide effective ventilation during the healing process while observing for scarring and tracheal stenosis. Generally, surgical repairs are considered when patients remain unstable or bridging the lesion is not technically feasible [9]. If the patient’s respiration is stable, positive pressure ventilation should be terminated [9]. A previous study showed that noninvasive positive pressure ventilatory support might be effective as transient treatment in patients with tracheal perforations [9]. However, positive pressure ventilation could not be discontinued in our patient because of disuse syndrome and cerebral palsy. As emergency treatment for tracheal perforation, we bridged the tracheal perforation with the cuffed tracheostomy tube, which successfully prevented the deterioration of mediastinitis and provided enough ventilation.

In conclusion, we observed unexpected hypotension because of pneumomediastinum and pneumothorax secondary to tracheal perforation in a pediatric patient. Our experience suggests that tracheal perforation should be considered in the differential diagnosis in pediatric patients with long-term tracheostomy tube placement who suddenly develop hypotension.

## Supplementary Information

**Additional file 1: Video 1**. Bronchoscopy and chest CT findings during hospitalization. Bronchoscopy findings just after the patient’s transfer to our hospital (0′ 02″–0′ 07″). Bronchoscopy findings after adjusting the position of the tracheostomy tube on the day of admission. A tracheal ulcer was observed on the posterior tracheal wall (0′ 07″–0′16″). Bronchoscopy findings on day 3. A tracheal perforation was confirmed on the posterior tracheal wall (0′ 16″–0′ 27″). Bronchoscopy findings after bridging the tracheal perforation with a cuffed tracheostomy tube (0′ 27″–0′ 32″). Pneumomediastinum, pneumothorax, and tracheal perforation were confirmed on thoracic CT (0′ 32″–0′ 37″). CT, computed tomography; A, anterior tracheal wall; P, posterior tracheal wall; L, left; R, right. The yellow and green arrows show pneumothorax and pneumomediastinum, respectively.

## Data Availability

Data not available due to ethical restrictions

## Abbreviation

CT: Computed tomography
